# (Dys)Prosody in Parkinson’s Disease: Effects of Medication and Disease Duration on Intonation and Prosodic Phrasing

**DOI:** 10.3390/brainsci11081100

**Published:** 2021-08-20

**Authors:** Sónia Frota, Marisa Cruz, Rita Cardoso, Isabel Guimarães, Joaquim J. Ferreira, Serge Pinto, Marina Vigário

**Affiliations:** 1 Center of Linguistics, School of Arts and Humanities, University of Lisbon, 1600-214 Lisbon, Portugal; marisac@edu.ulisboa.pt (M.C.); mvigario@edu.ulisboa.pt (M.V.); 2 CNS—Campus Neurológico, 2560-280 Torres Vedras, Portugal; ritasicardoso@gmail.com (R.C.); joaquimjferreira@gmail.com (J.J.F.); 3 Escola Superior de Saúde do Alcoitão, 2649-506 Alcabideche, Portugal; isabel.guimaraes@essa.scml.pt; 4 Instituto de Medicina Molecular João Lobo Antunes (IMM), Faculdade de Medicina, Universidade de Lisboa, 1649-028 Lisbon, Portugal; 5 Faculdade de Medicina, Universidade de Lisboa, 1649-028 Lisbon, Portugal; 6 Centre National de la Recherche Scientifique (CNRS), Laboratoire Parole et Langage (LPL), Aix Marseille Université, 13100 Aix-en-Provence, France; serge.pinto@lpl-aix.fr

**Keywords:** prosody, Parkinson’s disease, intonation, prosodic phrasing, prosodic phonology, intonational phonology, autosegmental–metrical (AM) approach, speech production, levodopa, disease duration

## Abstract

The phonology of prosody has received little attention in studies of motor speech disorders. The present study investigates the phonology of intonation (nuclear contours) and speech chunking (prosodic phrasing) in Parkinson’s disease (PD) as a function of medication intake and duration of the disease. Following methods of the prosodic and intonational phonology frameworks, we examined the ability of 30 PD patients to use intonation categories and prosodic phrasing structures in ways similar to 20 healthy controls to convey similar meanings. Speech data from PD patients were collected before and after a dopaminomimetic drug intake and were phonologically analyzed in relation to nuclear contours and intonational phrasing. Besides medication, disease duration and the presence of motor fluctuations were also factors included in the analyses. Overall, PD patients showed a decreased ability to use nuclear contours and prosodic phrasing. Medication improved intonation regardless of disease duration but did not help with dysprosodic phrasing. In turn, disease duration and motor fluctuations affected phrasing patterns but had no impact on intonation. Our study demonstrated that the phonology of prosody is impaired in PD, and prosodic categories and structures may be differently affected, with implications for the understanding of PD neurophysiology and therapy.

## 1. Introduction

Prosody is an essential component of language. In spoken languages, prosody is manifested by the use of phonetic features (such as pitch, duration, and intensity) to express meanings in ways that are structured by the language-particular system [1,2]. The prosodic grammar of a given language establishes the patterns of intonation, prominence, and speech chunking that characterize the language, as well as the prosodic form–meaning relations that convey linguistic and communicative meanings, such as finality, interrogativity, or highlighting of relevant information. Given that prosody plays a crucial role in human speech communication, prosodic impairments may seriously disrupt the ability to successfully interact with others. Prosodic disturbances have been described as a common feature in motor speech disorders [3,4], including Parkinson’s disease (PD). Although many aspects of prosody have been studied in PD patients, those related to the phonology of prosody have received little attention. It is largely unknown whether PD speakers use intonation categories and prosodic phrasing structures in ways similar to healthy speakers of the same language to convey similar meanings. The present study addresses this gap by investigating the phonology of key features of intonation and speech chunking in PD patients’ speech when compared to healthy speakers and as a function of medication intake and duration of the disease.

### 1.1. Prosody: Structure and Meaning

Prosody is used across languages to convey a broad range of linguistic functions. The present study focuses on phrase-level prosody, that is, on prosodic patterns beyond the individual word. Phrase-level prosody has long been described to convey three main functions [5]: (a) expressing a variety of communicative meanings, such as assertion, question, command, calling; (b) highlighting relevant information within a sentence by making it prosodically salient; (c) chunking the speech stream into meaningful units, thus facilitating the parsing of information. The following examples from European Portuguese illustrate the three linguistic functions. Functions (a) and (b) are illustrated in (1), with prosody encoding an assertion (1a), question (1b), and contrastive focus (1c); function (c) is illustrated in (2), with a major prosodic break in (2b) conveying a meaning that contrasts with (2a) where no break is found. The contrasts in (1) and (2) are conveyed by prosody, as the same word sequences are used. Languages vary in how intonation, prominence, and temporal features are combined to convey the different linguistic functions.

(1) a. A Maria vem. *Mary is coming.*
  b. A Maria vem? *Is Mary coming?*
  c. A Maria VEM. *Mary is coming (not leaving)*.
(2) a. A Maria bebeu o vinho. *Mary drank the wine.*
  b. A Maria, bebeu o vinho. *As for Mary, she drank the wine.*

The prosodic and intonational phonology frameworks [2,6,7] provide an approach that has been widely used to describe the intonation and prosodic structure of a variety of languages [8,9,10,11]. The autosegmental–metrical theory of intonational phonology describes intonation as a sequence of pitch events (high and low tones), which include pitch accents and boundary tones. Pitch accents mark prominent syllables, whereas boundary tones signal the edges of speech chunks, i.e., prosodic phrase edges. In the prosodic grammar of European Portuguese, the language of interest for the present study, only the edges of major prosodic phrases—called intonational phrases, are signaled by boundary tones [12]. The final pitch accent of an intonational phrase is usually the most prominent one and constitutes the nuclear contour together with the following boundary tone. Importantly, nuclear contours play a crucial role in the encoding of sentence types and pragmatic meanings, and each intonational phrase is characterized by a nuclear contour. In the autosegmental–metrical phonological description, pitch events and prosodic edges are annotated with a labeling system (a tone and break indices system, ToBI) that identifies the pitch accents, boundary tones, and relevant prosodic breaks. For example, the label L* stands for a low pitch accent, H*+L for a falling pitch accent with the H tone linked to the prominent syllable, and L+H* for a rising pitch accent with the H tone linked to the prominent syllable. L% represents a low intonational phrase boundary and LH% a rising boundary. Following the Portuguese ToBI (P-ToBI) system [13], the examples in (1) and (2) above have the phonological description respectively shown in (3) and (4), where intonational phrases are signaled by parentheses, and the nuclear word of each intonational phrase is underlined.

(3) a. (A Maria vem)  b. (A Maria vem) c. (A Maria VEM)
     H + L* L%       H + L* LH%     H* + L L%
(4) a. (A Maria bebeu o vinho) b. (A Maria)  (bebeu o vinho)
      H + L* L%      L* + H H%     H + L* L%

Therefore, nuclear contours and their pragmatic meanings are accounted for by the phonological contrast between pitch accents (e.g., H + L* versus H* + L) and/or boundary tones (e.g., L% versus LH%). Expressions such as parenthetical phrases or topics, in many languages, form an intonational phrase on their own [6,14] and thus trigger the chunking of the speech stream into several intonational phrases that demarcate the relevant, meaningful units. The present study uses the prosodic and intonational phonology frameworks to examine the features of nuclear contours and intonational phrasing in PD patients’ speech.

### 1.2. Prosody in Parkinson’s Disease

Parkinson’s disease (PD) is the second most frequent neurodegenerative disorder and the most common neurodegenerative movement disorder, affecting 1–1.5% of the population above 60 years of age [15,16,17,18]. PD is characterized by gross motor problems and motor speech impairment. Most PD patients (70–90%) develop hypokinetic dysarthria, a speech disorder that affects phonation, articulation, and prosody [19,20,21]. It has long been acknowledged that some of the most deviant characteristics of hypokinetic dysarthria are related to prosody; namely, monotony of pitch and intensity, reduced stress, variable speech rate, and inappropriate pauses [4,22,23,24]. Prosodic impairments in PD have been reported to affect the ability to convey linguistic and emotional meanings, as well as speech intelligibility, contributing to impaired communication, social isolation, and other psychosocial effects [22,25,26,27]. 

The characterization and assessment of prosody in PD have been predominantly based on perceptual impressions and auditory assessment (e.g., [28]), listeners’ judgments (e.g., [25,29,30]), self-reports (e.g., [31]), acoustic and articulatory measures (e.g., [32,33,34,35,36,37]), or a combination of these methods. On the basis of listeners’ judgments, prior work has shown that PD patients were less effective in conveying contrastive stress, the statement/question sentence type distinction, as well as boundary marking [25,29,38]. The many studies that have focused on the acoustic analysis of prosodic parameters, such as mean F0, F0 variability, overall trends in pitch and intensity, duration, speech, or articulatory rate, have demonstrated impairments in PD prosody. The speech of individuals with PD tends to be characterized by reduced F0 variation, narrower pitch range, lower intensity, reduced intensity range, shorter word duration, or reduced pause ratio (e.g., [32,33,35,39,40]). Few studies have investigated the phonetic parameters in a more functional way. In Tykalova et al. [35] and Thies et al. [36], the ability of PD patients to mark prosodic prominence was inspected. Tykalova et al. [35] found that despite a reduction of broad acoustic measures, PD patients could still convey emphasis. Looking at several acoustic and articulatory measures, Thies et al. [36] showed that, despite the reduced vowel space found in PD patients, they were able to express prominence using variations in F0, intensity, and vowel articulation in prominent positions (although modulated by medication intake, and less effectively than healthy speakers). 

In most studies so far, the measures considered have not been related to the linguistic categories or structural properties of prosody. In particular, the phonetic aspects of intonation measured, such as F0 variation or F0 range, have not been related to the phonological structure of intonation and the linguistic functions of pitch. Gili Fivela et al. [41] is the first study in this direction by assessing the correlation between acoustic measures related to linguistic prosodic contrasts and speech intelligibility in eight PD patients and four controls. Looking at acoustic measures of pitch and timing related to the expression of broad and corrective focus in Italian, they found no significant differences as a function of intelligibility, although the correlation between the acoustic measures and intelligibility ratings was stronger for sentences with corrective focus. While these preliminary data suggest that corrective focus might be more challenging for PD patients, the authors do not discuss whether or not the contrast between broad and corrective focus was successfully preserved in PD speech. Similarly, it is rare to find studies of PD prosody using phonological approaches that have proved successful for the description of linguistic prosody in healthy populations, such as the prosodic and intonational phonology frameworks [2,6]. Noteworthy exceptions include Mennen et al. [42] and Lowit and Kuschmann [43], who investigated the intonation of British English speakers with PD within the autosegmental–metrical theory of intonational phonology. Mennen et al. [42] observed the read speech of two individuals with PD and two age-matched controls and found that PD patients used fewer pitch accents per intonational phrase and produced shorter phrases than healthy controls. Lowit and Kuschmann [43] examined the spontaneous speech of eight PD patients and found that the prevalence and frequency of pitch accents and boundary tones differed from controls, with a higher rate of pitch accents, shorter phrases, and more phrase-initial high boundary tones. However, both studies reported that the inventory of pitch accents and boundary tones in PD patients and healthy controls were similar, but none specifically investigated whether sentence type or contrastive focus were prosodically conveyed as expected, nor the phonological adequacy of the intonational phrasing. Lowit and Kuschmann [43] point out the need to conduct more controlled research. Thus, the ability of PD patients to use the prosodic categories and structure of the grammar of their native language remains largely unknown.

Importantly, prosodic impairments in PD patients have been reported not only for expressive but also for receptive prosody [22,44,45,46,47]. For example, a decreased ability at the identification of utterance prosody and impaired comprehension of prosody and lexical stress have been found in individuals with PD when compared to controls. However, other studies have reported opposite results, with PD patients and controls performing similarly in the perception and comprehension of five communicative functions of Dutch prosody [48] or in a speech segmentation task [49]. It is thus an open question whether the prosodic categories and their respective linguistic functions and meanings are preserved in PD.

### 1.3. Dopaminergic Treatment, Disease Duration, and Prosody

Motor problems in PD are caused by deficits in dopaminergic brain systems [50]. Dopaminergic drugs, such as levodopa, have been reported as the most common and efficacious treatment for PD [51]. The effects of dopaminergic treatment on speech prosody seem to be variable and unclear [23,52,53]. Several studies suggest that levodopa affects the realization of some prosodic parameters. Pinho et al. [54] reported that at the acoustic level, levodopa intake has been associated with modifications in F0 but not vocal intensity. In an acoustic study examining the effect of levodopa on the production of four different sentence modalities (expressing certainty, doubt, assertion, and interrogativity), Azevedo et al. [55] reported an effect of medication only at the level of syllabic duration, with shorter syllables, while intensity and F0 remained unchanged. Thies et al. [37] investigated prominence marking in PD patients considering the effect of medication. Acoustic measures and articulatory data of tongue movements were analyzed. Three prominence marking conditions were considered: prominence marking of given information (unaccented), broad focus, and contrastive focus. They reported that PD patients are able to distinguish the three conditions by producing longer vowel durations and higher F0 rises in broad, and more so, in contrastive focus. Importantly, Thies and colleagues also show that the effects of drug intake may depend on the kind of measure considered, as they found an effect of medication intake in the agility of tongue movement in the articulatory measure, which however had no counterpart in the acoustic measures, namely, in the acoustic vowel space. Fewer studies investigated the prosodic effects of dopaminergic medication beyond speech production and acoustic or articulatory measures. In Cameron et al. [56], the performance of PD patients in a task where participants had to determine whether two rhythms were the same or different was investigated. Cameron et al. [56] found that the impact of medication depended on rhythm type, with medication increasing sensitivity to changes in simple, beat-based rhythms and decreasing sensitivity to changes in complex, non-beat-based rhythms. This might suggest improved performance for less complex prosodic patterns and worse performance for more complex prosodic patterns. 

As noted above, the effects of levodopa on speech prosody seem inconclusive, depending on the measure and the task, among other factors. The effects of long-term use of medication are also inconclusive and complex, with some studies reporting beneficial effects mostly in the early years of the disease and others in more advanced patients with PD [39,57,58]. The lack of effectiveness of levodopa and the severity of the disease may result in motor fluctuations, which also affect speech [59]. Independently of medication, disease duration also seems to be reflected in dysprosody. Reduced F0 variability that tends to deteriorate with disease duration is sometimes described [34]. Variation in speech rate has also been found, with acceleration at the beginning of the disease and slower rates at more advanced years [33]. However, Skodda et al. [40] found no significant differences in F0 variability and pause ratio as a function of progression of the disease, and a similar finding was reported in Bowen et al. [60] relative to disease duration.

Crucially, to the best of our knowledge, the effects of dopaminergic intake, or of disease duration, on the use of prosodic categories and their phonological patterns in PD patients’ speech, namely, nuclear contours and intonational phrasing, have not yet been investigated.

### 1.4. The Present Study

The aim of the present study is to examine the phonology of intonation and speech chunking in PD patients’ speech and as a function of medication intake and duration of the disease. Specifically, we investigate the features of nuclear contours and intonational phrasing to establish the ability of PD patients to use the prosodic categories and structures of the native language to convey their respective linguistic functions and meanings. We asked three main questions: (i) How is intonation used to express sentence types and pragmatic meanings (e.g., statements, questions, contrastive focus)? (ii) How is prosodic phrasing used to chunk the speech stream into meaningful linguistic units (e.g., parenthetical, topic phrases)? (iii) How do medication and disease duration affect intonation and prosodic phrasing? To answer these questions, the prosody of PD patients’ speech, and healthy controls, was analyzed following the methods of the prosodic and intonational phonology frameworks to examine the features of nuclear contours and intonational phrasing. As previous research has not addressed the phonology of prosody, including form–meaning relations that convey linguistic and communicative meanings, the hypotheses driving this study were motivated by the general findings on the characteristics of prosody in PD reviewed in Section 1.2 and Section 1.3. 

If prosodic disturbances in PD affect prosodic categories and structures, patients with PD will show a decreased ability to use nuclear contours and prosodic phrasing. By contrast, if prosodic disturbances in PD are only a matter of phonetic realization (e.g., degree of pitch movement), it is expected that prosodic categories and structures are mostly preserved.

If prosodic disturbances in PD are found to affect prosodic categories and structures, medication is expected to improve PD patients’ ability to use nuclear contours and prosodic phrasing, given the previously described general gains on motor dysfunction and speech. By contrast, the duration of the disease, as well as the presence of motor fluctuations, are expected to negatively impact PD patients’ ability to use the prosodic patterns of the language. 

## 2. Materials and Methods

### 2.1. Participants and Assessments

Thirty patients with idiopathic Parkinson’s disease (15 male, 15 female) aged between 40 and 82 years participated in the study, together with 20 healthy controls age- and gender-matched (Table 1). The participants were part of a larger pool of participants studied within the FraLusoPark project and recruited at the Movement Disorders Unit, Hospital de Santa Maria, Lisbon, and Campus Neurológico Sénior (CNS), Torres Vedras, Portugal [57]. For the current study, inclusion and exclusion criteria were as follows: the diagnosis of idiopathic PD (according to the UK Parkinson’s Disease Brain Bank Criteria [61]) for patients, and the absence of any neurological, psychiatric, or behavioral pathology for controls. In addition, all participants had the standard variety of European Portuguese (SEP) as the native language (i.e., all participants lived in the large Lisbon area for more than 10 years, according to the criteria of the Interactive Atlas of the Prosody of Portuguese [62]). None of the patients had cognitive deficits, severe depression, or dementia (assessed with the Montreal Cognitive Assessment (MoCA [63]), and the Clinical Global Impressions scale (CGI [64]); for the full list of inclusion and exclusion criteria within the FraLusoPark project, see [57]). There was no significant difference in age between patients and controls (*t*(48) = −1.195, *p* > 0.1, *d* = 0.345).

Disease duration varied between less than 1 year and 23 years, and the severity of dysarthria also varied (assessed with the Frenchay Dysarthria Assessment (FDA-2) [65]), with some of the patients showing motor fluctuations. All patients were undergoing levodopa therapy and had never been treated with deep brain stimulation. General motor performance was assessed using part III of the MDS Revision of the Unified Parkinson’s Disease Rating Scale Portuguese Translation (MDS-UPDRS [66]). Motor scores were obtained before (Med-OFF) and after (Med-ON) a dopaminomimetic drug intake (see Section 2.2). The scores before and after medication differed significantly (*t*(29) = 6.369, *p* = 0.000; *d* = 1.16). Levodopa equivalent doses taken in the Med-ON condition are also reported in Table 1 (LED reports follow [67,68]). 

The study was approved by the Ethics Committee of the Lisbon Academic Medical Centre (project reference number 239-14). 

### 2.2. Materials and Procedure

A set of speech materials was designed to elicit specific prosodic patterns. Materials consisted of 40 items, including diverse sentence types and pragmatic meanings, as well as diverse prosodic phrasings. The five sentence types and pragmatic meanings chosen are conveyed by distinct intonation patterns, in particular by specific nuclear contours realized on the most prominent word of the utterance (see Section 1.1). The nuclear contours for the five sentence types and pragmatic meanings, according to the prosodic grammar of SEP, are given in Table 2.

In addition, the speech materials include four types of items that trigger the prosodic chunking of the speech stream into several major prosodic phrases, called intonational phrases [12]. Intonational phrase breaks require the presence of a boundary tone and are commonly signaled by a pitch movement, pre-boundary lengthening, and/or a pause. The expected prosodic phrasing for the four types of multi-phrase items, according to the prosodic grammar of SEP, is illustrated in (5)–(8), where parentheses indicate chunking into intonational phrases.

(5) Topic phrase: (Às alunas) (os amigos ofereceram rosas)
        *To-the students the friends gave roses*
(6) Parenthetical phrase: (O Álvaro) (antes de partir) (falou com os amigos)
        *Álvaro   before leaving  talked to his friends*
(7) Enumeration: (Gosto de maçã) (banana) (uvas) (laranja) (e tangerina)
        *(I) like apple   banana grape  orange and tangerine*
(8) Related sentences: (O músico compôs uma cantiga) (Ela inspirou-o)
        *The musician wrote a song   She inspired him*

Most items were preceded by a context, presented by the researcher, and were read by the participant in response to the context, as illustrated in (9). The context elicited target utterances with the intended sentence type and pragmatic meaning. The 40 items and the contexts are listed in Appendix A.

(9) Context: Quer que a Marina venha para que o jantar possa ser servido. Chame-a.
  *You want Marina to come so that dinner can be served. So you call her.*
  Target: Marina!

The 40 items were divided into two sets of 20 items. The two sets have differences in the lexicon used but are similar in all the other properties, namely, in their prosodic characteristics (such as the prosodic phrasing patterns and prosodic features of the nuclear words). PD participants completed the speech task twice, each time using a different set of items, before and after a dopaminomimetic drug intake. The order was fixed and started in the medication-OFF condition (OFF) followed by the medication-ON condition (ON). The OFF condition implied at least 12 h after withdrawal of all anti-Parkinsonian drugs. The ON condition implied at least 1 h after the administration of the usual medication [57]. Healthy control participants completed the speech task only once, using one of the sets of items. 

The speech recordings took place during a session with a speech therapist in which the participant (patient or control) completed a series of speaking tasks as part of a larger protocol [57]. The order of the tasks was fixed across participants. The recordings took place in a quiet room, using a Marantz PMD recorder and a DPA headset microphone. Each item was produced once, and only utterances that were incorrectly produced (i.e., by mistakenly mispronouncing a word) were repeated. In total, 1600 tokens were obtained: 1200 tokens for PD participants (20 items × 2 medication conditions × 30 speakers) and 400 tokens for controls (20 items × 20 speakers).

### 2.3. Prosodic Analysis

**Figure 1:**
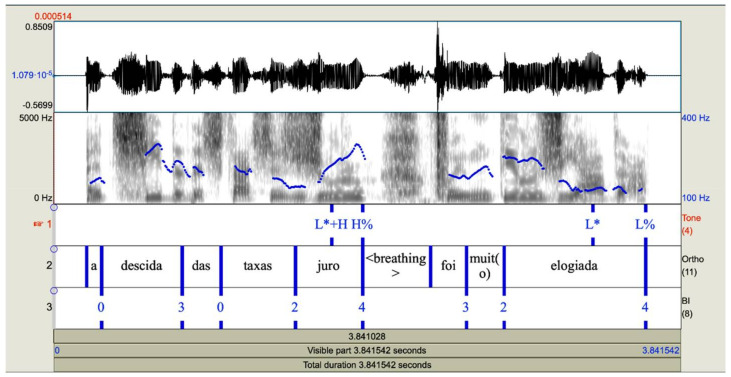
Textgrid with three tiers for annotation: tone, orthography, and break indices.

The speech signal was recorded at 44.1 kHz, and later downsampled at 22.50 kHz. The prosodic analysis was conducted using Praat [70], and the P-ToBI labeling system [12,13]. For each token, a textgrid was created in Praat with three tiers for annotation: a tone tier, for the analysis of intonation; an orthography tier, where each token is segmented into orthographic words and silences/pauses; and a break indices tier, for the analysis of prosodic phrasing (Figure 1). The two key tiers for the purposes of the current study are the tone tier and the break indices tier. 

Prosodic analysis and related prosodic annotation were performed on the basis of the analysis of the pitch curve, spectrogram, and soundwave, combined with perception. Two of the authors, who are experts in prosody, independently annotated each token. Differences in annotation were either solved between the two authors or with the intervention of a third author (also an expert in prosody). Thirteen tokens had to be excluded due to poor sound quality or incorrect production by the participant (for example, use of different words or sentence structure that had an impact on prosody, as in the calling sentence ‘Ó Marina’ *Hey Marina*, instead of ‘Marina’, where the word ‘Ó’ already expresses the function/meaning of calling). A total of 1587 tokens were included in the analysis: 1193 for PD participants and 394 for controls. 

#### 2.3.1. Intonation: Sentence Type and Pragmatic Meaning

The nuclear contours for the five sentence types and pragmatic meanings under analysis were inspected on the basis of the phonological annotation in the tone tier. The analysis involved three steps. First, for each participant, the nuclear contour produced in each token was compared to the expected intonation pattern according to the prosodic grammar of SEP, following P-ToBI (Table 2). Matching nuclear contours were classified as ‘non-deviant’ and non-matching nuclear contours as ‘deviant’. For example, the matching nuclear contour for a yes–no question is a falling–rising pitch pattern (H+L* LH% in the P-ToBI labeling scheme). Therefore, any of the following pitch patterns would be deviant: rising (e.g., L* H%), rising–falling (e.g., L*+H L%), and falling (e.g., H+L* L%). However, not all tonal patterns are equally deviant. The rising pattern is more similar to the matching pattern, given that it simplifies the contour but preserves the final rise that expresses interrogativity The falling pattern, by contrast, is the most deviant one, given that it includes no rising pitch and conveys a declarative meaning. Four degrees of ‘deviance’ (D) were considered, as defined in Table 3. 

In the second step, the proportion of each type of D in the tokens of each participant for each sentence type was computed. For PD participants, the tokens produced in the OFF and ON conditions were analyzed separately. Finally, in the third step, the highest D was used to place the participant in a scale where ‘1′ means non-deviant from the prosody of the native language and ‘−1′ means completely deviant from the prosody of the native language. Participant placement was according to the correspondence table in Table 4. The value in the scale for each healthy participant and for each patient in the OFF and ON conditions was used as an intonation index in the statistical analysis.

#### 2.3.2. Prosodic Phrasing

**Figure 2:**
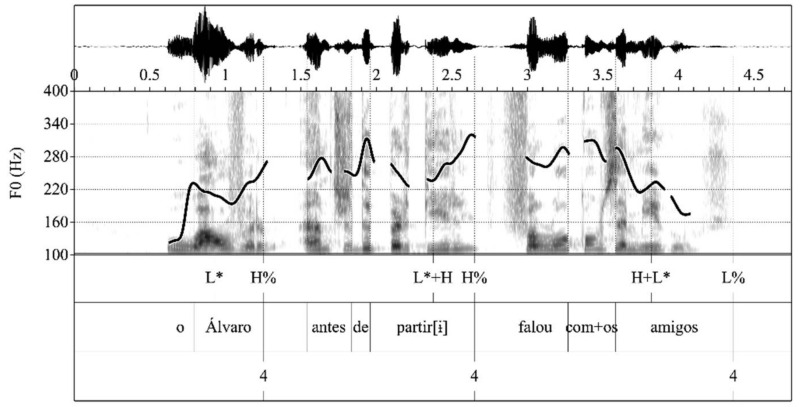
Expected intonational phrase breaks produced by a PD patient.

**Figure 3:**
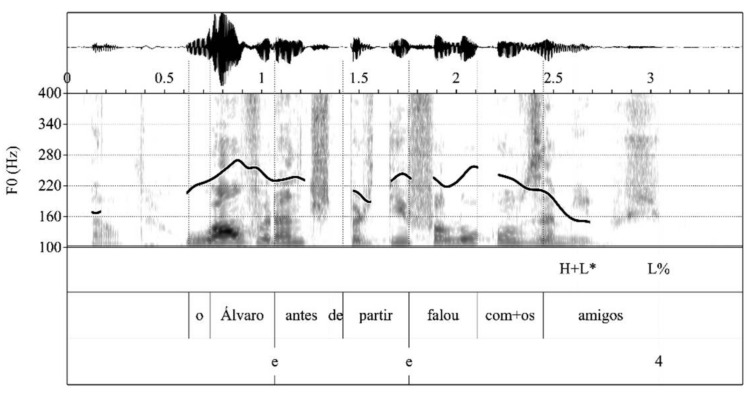
Expected intonational phrase breaks not produced by a PD patient.

The prosodic phrasing of the four types of items that trigger the prosodic chunking of the speech stream into intonational phrases was examined on the basis of the annotation in the break indices tier. Following the P-ToBI annotation conventions [13], intonational phrase breaks were marked with break index ‘4’. In the current study, expected intonational phrase breaks that were not produced were marked with ‘e’. The analysis involved three steps. First, for each participant, the prosodic chunking into intonational phrases produced in each token was compared to the expected prosodic phrasing according to the prosodic grammar of SEP (see (1)–(4) above). The number of expected breaks produced (‘4′) was computed, as well as the number of expected breaks that were not produced (‘e’). For the computation of expected breaks, the final break at the end of each production was discarded (e.g., the third ‘4′ in Figure 2 and Figure 3). Figure 2 and Figure 3, respectively, illustrate renditions of the item in (2) with and without the expected utterance internal intonational phrase breaks. 

In the second step, the proportion of expected breaks produced and the proportion of expected breaks not produced were computed per participant, considering all the breaks that were produced plus those that could have been produced. Produced intonational breaks that were misplaced in the speech stream were quite infrequent, and thus their proportion was not computed. For PD participants, the tokens produced in the OFF and ON conditions were analyzed separately. 

Finally, in the third step, the proportion of expected breaks produced (‘4’) and the proportion of expected breaks not produced (‘e’) was used to place the participant in a scale where ‘1’ means non-deviant from the prosody of the native language and ‘−1’ means completely deviant from the prosody of the native language: for example, 100% production of ‘4’ is equivalent to ‘1’, 50% to ‘0’, and 0% to ‘−1’; by contrast, 100% of ‘e’ equals ‘−1’, 50% is ‘0’, and 0% is ‘1’. The mean value of the two scales (‘4’ + ‘e’/2) for each healthy participant and for each patient in the OFF and ON conditions was used as a prosodic phrasing index in the statistical analysis.

### 2.4. Statistical Analysis

The intonation index and the prosodic phrasing index were analyzed separately. First, we focused on differences between healthy controls (C) and groups of patients according to disease duration. Following the criteria defined in Pinto et al. (2016), we considered three subgroups of patients (Table 5): group 1 (G1) with a disease duration between 0 and 3 years (and stages 1 and 1.5 in the modified Hoehn and Yahr scale); group 2 (G2) with a disease duration between 4 and 9 years (and stages 2 and 3 in the modified Hoehn and Yahr scale), and group 3 (G3) with a disease duration of 10 years or more (stages 4 and 5 in the modified Hoehn and Yahr scale). There was no significant difference in age between groups (*F*(2, 27) = 2.08, *p* > 0.1, η2 = 0.13). Twenty observations for each group (C, G1, G2, and G3) were compared to investigate the effect of disease duration on intonation and prosodic phrasing, by means of a One-Way ANOVA or a Kruskal–Wallis test, in case conditions for running the ANOVAs were not met. Then, we focused on the groups of patients to investigate the effects of medication and disease duration by means of a mixed-ANOVA. If a main effect of medication (or an interaction) was found, the Wilcoxon Signed Ranks Test was used to further examine the effects of medication within each group of patients.

Finally, the patients’ data were further analyzed by means of generalized linear mixed models in SPSS (IBM SPSS Statistics, version 26.0), with the goal of examining effects of medication, disease duration, and presence of motor fluctuations while considering individual variation. We controlled for participants in the random effect structure, used a Satterthwaite approximation to account for differences, and robust estimation of the covariances to account for small sample sizes. Fixed effects were medication (OFF, ON, as within-subject factor), motor fluctuations (yes, no), and disease duration (as a continuous variable). 

## 3. Results

### 3.1. Intonation Results

**Figure 4:**
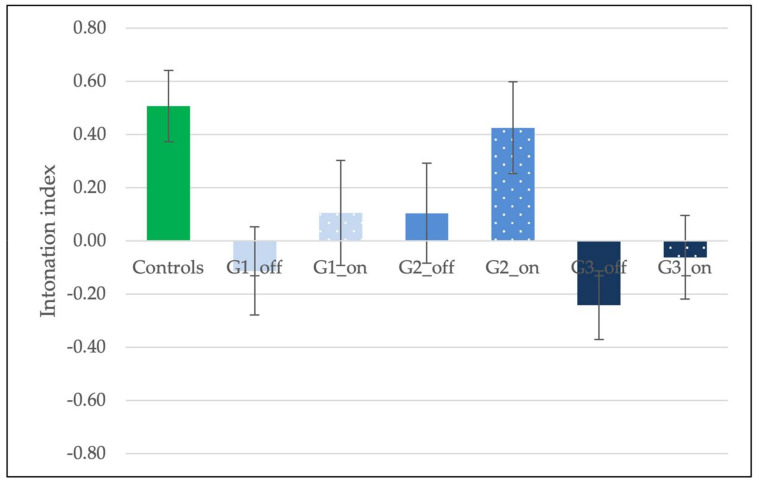
Average intonation index across groups (Controls, and the three groups of patients, G1, G2, and G3) and medication conditions (medication-OFF and medication-ON). Error bars indicate the standard error of the mean (±1).

Figure 4 shows the average intonation index across groups and medication conditions. First, the performance of healthy controls and the three groups of PD patients arranged according to disease duration [57] was analyzed. A One-Way ANOVA revealed a significant effect of Group (*F*(3, 76) = 5.54, *p* = 0.002, η2 = 0.18). Pairwise comparisons (Bonferroni-controlled) showed no differences between groups of patients (*p*s > 0.1), while controls differed from both G1 (*p* = 0.02) and G3 (*p* = 0.002), but not G2 (*p* > 0.1). Thus, the intonation index of PD patients was not significantly affected by disease duration, and controls are generally better than patients, especially in the case of G1 and G3. 

We then examined the effects of medication and disease duration focusing on the three groups of patients. A mixed-ANOVA showed an effect of medication (*F*(1, 27) = 4.59, *p* = 0.04, ηp2 = 0.15), but no effect of disease duration (*F*(1, 27) = 2.30, *p* > 0.1, ηp2 = 0.02) and no interaction (*F*(2, 27) = 0.14, *p* > 0.1, ηp2 = 0.01). Patients were generally better in the ON condition (M = 0.16, SE = 0.10) than in the OFF condition (M = −0.08, SE = 0.09). A Wilcoxon Signed Ranks Test (one-tailed) revealed that the medication-OFF and -ON conditions did not differ within groups, although all the groups of patients improved in the ON condition, as depicted in Figure 4 (G1 OFF M = −0.11, G1 ON M = 0.11, *Z* = −0.87, *p* > 0.1, *r* = −0.27; G2 OFF M = 0.10, G2 ON M = 0.43, *Z* = −1.38, *p* = 0.08, *r* = −0.44; G3 OFF M = −0.24, G3 ON M = −0.06, *Z* = −0.82, *p* > 0.1, *r* = −0.26). 

To further examine PD patients’ production of nuclear contours, a generalized linear mixed model was run with the participant as the random effect and medication (ON, OFF), motor fluctuations (yes, no), and disease duration as fixed effects. Disease duration was included in the model as a continuous variable. The model was computed with and without the interaction term. Inclusion of the interaction term did not improve the model, and the model without the interaction was superior (based on the comparison of the −2 log likelihood information criterion). We thus report the simpler model. There was a significant effect of medication (patients were better in the ON than in the OFF condition, *F*(1, 56) = 4.87, *p* = 0.03; *β* = −0.24, SE = 0.11, *t* = −2.21, *p* = 0.03), with no effect of disease duration (*F*(1, 56) = 0.72, *p* > 0.1) or motor fluctuations (*F*(1, 56) = 0.20, *p* > 0.1). The linear mixed model analysis confirmed the effect of levodopa medication on the intonation index, as well as the absence of an effect of disease duration. In addition, it showed that the presence of motor fluctuations had no impact on the intonation index.

### 3.2. Prosodic Phrasing Results

**Figure 5:**
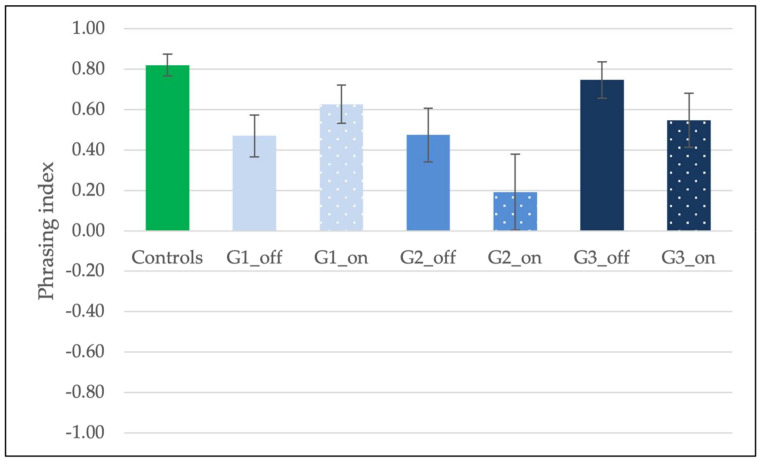
Average prosodic phrasing index across groups (Controls, and the three groups of patients, G1, G2, and G3) and medication conditions (medication-OFF and medication-ON). Error bars indicate the standard error of the mean (±1).

Figure 5 shows the average prosodic phrasing index across groups and medication conditions. First the performance of healthy controls and the three groups of patients arranged according to disease duration was analyzed. A Kruskal–Wallis test revealed significant differences between groups (*H*(3) = 15.62, *p* = 0.001). Mann–Whitney tests (one-tailed) showed that healthy controls were better than all groups of patients (G1, *U* = 89.00, *z* = −3.04, *p* = 0.001, *r* = −0.48; G2 *U* = 71.00, *z* = −3.52, *p* < 0.001, *r* = −0.56; G3 *U* = 142.00, *z* = −1.59, *p* = 0.05, *r* = −0.25). In addition, G2 differed from G3 (*U* = 124.00, *z* = −2.06, *p* = 0.02, *r* = −0.33), with G3 showing better performance. No other differences between groups of patients were found (*p*s > 0.1).

Then, focusing on the three patient groups, the effects of medication and disease duration were inspected. A mixed-ANOVA showed no effect of medication (*F*(1, 27) = 1.88, *p* > 0.1, ηp2 = 0.07), no effect of disease duration (*F*(1, 27) = 2.18, *p* > 0.1, ηp2 = 0.14), and a borderline interaction between the two factors (*F*(2, 27) = 2.89, *p* = 0.07, ηp2 = 0.18). The borderline interaction reflects the pattern of improvement from OFF to ON in G1, whereas G2 and G3 show the reverse pattern with the ON condition yielding a lower phrasing index (Figure 5). 

We further examined PD patients’ production of intonational phrase breaks by computing a generalized linear mixed model with the participant as the random effect and medication (ON, OFF), motor fluctuations (yes, no), and disease duration (continuous variable) as fixed effects. Again, the inclusion of the interaction term did not improve the model, and the model without the interaction was superior (based on the comparison of the −2 log likelihood information criterion). Therefore, the results of the simpler model are reported. The model showed a significant effect of motor fluctuations (patients without motor fluctuations were better at phrasing than patients with motor fluctuations, *F*(1, 56) = 4.68, *p* = 0.03; *β* = 0.31, SE = 0.15, *t* = 2.16, *p* = 0.03) and of disease duration (*F*(1, 56) = 4.31, *p* = 0.04; *β* = 0.02, SE = 0.01, *t* = 2.07, *p* = 0.04), with no effect of medication (*F*(1, 56) = 1.66, *p* > 0.1). Disease duration showed a U-shaped curve, with better performance in shorter and longer durations than between 5 and 10 years of disease duration. The linear mixed model analysis confirmed that levodopa medication had no effect on the prosodic phrasing index while revealing effects of both disease duration and motor fluctuations.

## 4. Discussion

The aim of the present study was to investigate the phonology of intonation and speech chunking in PD patients’ speech and as a function of medication intake and duration of the disease. Specifically, we examined the features of nuclear contours and intonational phrasing to establish the ability of PD patients to use the prosodic categories and structures of the native language to convey their respective linguistic functions and meanings. To this end, we used the prosodic and intonational phonology frameworks, and in particular the autosegmental–metrical approach [2,6,7], as tools to describe prosody in PD. 

One of the main findings of the current study was that PD patients show a decreased ability to use nuclear contours and prosodic phrasing when compared to healthy controls. Therefore, prosodic impairments in PD are not only a matter of phonetic realization, and affect prosodic categories (nuclear contours) and structures (prosodic phrasing). Although there is no one-to-one mapping between phonetics (variation in pitch, intensity, or duration) and the phonological categories of prosody, this result suggests that previous reports of deviant phonetic parameters (e.g., [4,22,23,24,32,33,35,39,40]) might at least partially reflect changes in the phonology of prosody. Findings from two prior studies that investigated the intonation of British English speakers with PD within the autosegmental–metrical theory of intonational phonology [42,43] had already suggested differences in phonological aspects of intonation and speech chunking, namely, in the type and distribution of pitch accent categories, and in the size of prosodic phrases. However, those studies focused on a different language, included a very small number of participants, and did not investigate whether sentence type and pragmatic meaning, or intonational phrasing, were prosodically conveyed as expected. The current study has offered new evidence that PD speakers show a diminished ability to use intonation categories and prosodic phrasing structures in ways similar to healthy speakers of the same language to convey similar meanings.

Another main finding of our study was that medication and disease duration differently affected the categories of intonation and the patterns of prosodic phrasing. After medication intake, PD patients showed an improved ability to use nuclear contours to convey sentence types and pragmatic meanings, as expected. This is in line with previous reports on the beneficial effects of dopaminergic treatment on pitch variability [52]. However, both the duration of the disease and the presence/absence of motor fluctuations had no impact on the categories of intonation. The patterns of prosodic phrasing, by contrast, were not affected by medication but were affected by disease duration and the presence of motor fluctuations. The latter led to more deviant patterns of prosodic phrasing, as could be expected in line with more severe manifestations of the disease [57,59]. The effect of disease duration was unexpected, with better prosodic phrasing in longer (10 years or more) than intermediate (between 5 and 10 years) disease durations. The better prosodic phrasing found in the longer disease durations might relate to the decrease in speech rate that has been reported throughout the course of the disease [37], which might facilitate the chunking of utterances with small- and medium-sized intonational phrases (i.e., one to three lexical words), such as the ones used in our materials. Future studies using utterances with more varying phrase lengths, and thus posing a higher phrasing challenge under decreased speech rate conditions, are needed to further determine how prosodic phrasing patterns evolve with disease duration. Moreover, although no effect of levodopa on prosodic phrasing was found in the current study, there was a tendency for improvement in the medication-ON condition only in the early stages of the disease.

The differences between intonation and prosodic phrasing concerning the effects of medication and disease duration were not expected. Previous work on PD prosody does not offer an explanation for this finding. Nuclear contours and phrasing patterns, however, are distinct phonological mechanisms. Nuclear contours are formed by tonal categories included in an inventory of such categories, the intonational lexicon, where form–meaning relations are established [2,71]. The choice between such intonation categories, e.g., the choice between H+L* L%, which conveys a statement, and H+L* LH%, which conveys a question in European Portuguese, is thus a paradigmatic choice dependent on the intended linguistic and communicative meanings. Unlike in the case of nuclear contours, the presence of a phrasing boundary crucially relies on other phrasing boundaries, that is, on the phrasing patterns of the utterance. In other words, prosodic phrasing is determined syntagmatically, taking into account the speech stretch before and after a phrasing boundary. In addition, prosodic phrasing also takes into account the mapping between different kinds of linguistic structures, namely, the morphosyntactic structure (the structure of words and their combination to form a sentence) and the phonological sound structure [72]. For example, the mapping rules for European Portuguese establish that parenthetical phrases are signaled by intonational phrase breaks [12]. Interestingly, besides the complexity of the neural networks for prosody described in recent research, it has been suggested that the processing of pitch contour differences between statements and questions involves both the ventral and dorsal pathways in the right hemisphere [73], while the processing of prosodic phrasing involves the right fronto-temporal and dorsal networks, together with inter-hemispheric pathways to map morphosyntactic and phonological structures [74]. The different effects of medication and disease duration on nuclear contours and prosodic phrasing found in the current study raise new questions that future research on PD prosody should address to better understand the mechanisms underlying prosodic impairments and inform therapeutic planning. 

The novel finding that prosodic impairments in PD affect prosodic categories and structures, such as nuclear contours and phrasing patterns, raises the question of the importance of the native language. General similar effects on acoustic measures across languages have been recently reported [75]. However, it is known that different languages may prosodically encode linguistic information in different ways [2,7,8,9,10,11]. For example, focus or the highlighting of relevant information is encoded in different ways across languages [14,76], with some languages crucially using pitch accent type distinctions (as European Portuguese), or prominence placement (as English), or prosodic phrasing (as Korean). In some languages, there is no prosodic encoding of focus (as in Ambonese Malay [77]). Therefore, the impact of prosodic impairments in PD patients at the linguistic and communicative levels may vary depending on the patients’ native language. Similarly, the effects of medication might also vary. Although the language-particular factor has been recently acknowledged [23,57,78], studies on PD prosody in different languages are lacking, especially studies on the phonology of prosody. Such studies are required to inform more language-driven approaches to the treatment of prosodic impairments in PD.

The promising findings of the current study open new avenues for further research. In particular, future research should investigate nuclear contours and phrasing patterns in PD patients’ speech in other languages, using similar methodological approaches. Another direction for future research is extending the account of prosodic and intonational phonology in PD to include non-nuclear pitch events and other types of prosodic breaks/levels of prosodic phrasing involved in the encoding of linguistic and communicative meanings, thus further exploring the potential of the prosodic and intonational phonology frameworks to offer descriptions of impaired prosody. 

## 5. Conclusions

The current study demonstrated that the phonology of key features of intonation and speech chunking in PD patients’ speech is impaired, with consequences for the expression of linguistic and communicative meanings. Furthermore, medication improved the ability to express sentence types and pragmatic meanings through intonation, regardless of disease duration, but did not help with dysprosodic speech chunking, which evolved differently. In conclusion, prosody is a complex system, and different prosodic categories and structures may be differently affected in PD, with implications for the understanding of PD neurophysiology and therapy.

## Figures and Tables

**Table 1:** Participants’ demographic characteristics. For patients, disease duration, MDS-UPDRS scores, and levodopa equivalent doses (LED) taken in the Med-ON condition are given.

Patients							Healthy Controls		
ID	Gender	Age	Disease Duration (Years)	MDS-UPDRS III (OFF)	MDS-UPDRS III (ON)	LED	ID	Gender	Age
PD01	M	65	3	21	19	100	C01	F	74
PD02	F	58	1	35	30	--- *	C02	M	59
PD03	F	59	3	29	23	370	C03	F	51
PD04	M	59	3	32	29	150	C04	F	74
PD05	F	71	1	23	16	80	C05	M	73
PD06	F	80	2	46	36	100	C06	F	63
PD07	F	52	2	29	23	260	C07	M	64
PD08	F	70	<1	43	43	--- *	C08	F	59
PD09	M	82	3	70	65	125	C09	F	68
PD10	M	67	1	37	30	100	C10	F	50
PD11	F	40	5	38	19	500	C11	M	62
PD12	F	64	4	18	11	420	C12	F	43
PD13	M	72	4	34	22	100	C13	M	65
PD14	M	72	5	48	37	290	C14	M	62
PD15	F	60	4	64	43	--- *	C15	M	66
PD16	M	73	7	26	22	--- *	C16	M	54
PD17	F	54	8	29	24	300	C17	F	44
PD18	F	41	6	43	15	360	C18	F	63
PD19	F	56	7	27	24	100	C19	M	52
PD20	M	48	8	48	38	416	C20	M	54
PD21	M	79	15	75	43	200			
PD22	M	70	16	60	40	200			
PD23	F	65	10	30	21	240			
PD24	F	74	23	26	27	--- *			
PD25	M	53	15	56	49	570			
PD26	F	61	13	38	19	100			
PD27	M	63	13	64	43	460			
PD28	M	52	15	35	27	626			
PD29	M	73	16	28	29	530			
PD30	M	73	14	34	28	200			
Mean	---	63.4	7.6	39.5	29.8	275.9	---	---	60.0
SD	---	(10.9)	(6.0)	(15.1)	(11.8)	(170.3)	---	---	(9.2)

* Due to missing data, LED could not be calculated.

**Table 2:** Nuclear contours: prosodic labels following P-ToBI, phonetic realization, and context/meaning/usage. The grey box signals the stressed syllable of the nuclear word. Adapted from [12,69].

Labels	Realization	Context/Meaning/Usage
H+L* L%	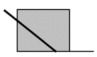	Broad focus statements
H*+L L%	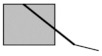	Contrastive focus
H*+L L%	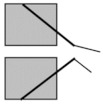	Imperative sentences (commands)
L*+H L%		
H+L* LH%	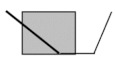	Interrogative sentences (yes–no questions)
L+H* !H%	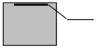	Calling sentences (first call)

**Table 3:** Analysis of nuclear contours: definition of degrees of deviance relative to the prosodic grammar of the native language.

Deviance	Broad Focus Statement	Contrastive Focus	Command	Yes–No Question	Calling
D1 (non-deviant)	H+L* L%	H*+L L%	H*+L L%/L*+H L%	Falling–rising	L+H* !H%
D2 (less deviant)	L* L%	¡H+H* L%	---	All–rising	---
D3 (more deviant)	---	H+L* L%	L* L%	Rising–falling	L+H* L%
D4 (highly deviant)	---	H* !H%	---	All–falling	H*+L !H%/L*+H H%

**Table 4:** Correspondence table for the computation of the intonation index.

Deviance	Position	Equation
D1	1 to 0.5	0.5 + D*0.5/100
D2	0.5 to 0	0.5 − D*0.5/100
D3	0 to −0.5	0 + D* − 0.5/100
D4	−0.5 to −1	−0.5 + D* − 0.5/100

**Table 5:** Groups of patients: gender, mean, and SD values for age and disease duration.

Patients			
Group	Gender	Age	Disease Duration (Years)
G1	6F/4M	66.30 (9.73)	1.95 (1.01)
G2	6F/4M	58.00 (12.43)	5.80 (1.62)
G3	3F/7M	66.30 (9.10)	15.00 (3.33)

## Data Availability

The raw data supporting the conclusions of this article will be made available by the authors, without undue reservation, on request to the corresponding author.

## Appendix A

### Appendix A.1. Set 1

1. A loura cantava uma melodia maravilhosa de Espanha.
  ‘The blond girl was singing a wonderful melody from Spain.’
2. (C: O senhor/A senhora saiu de casa e viu a rua molhada. Então, perguntou se choveu:) Choveu?
  ‘(C: You went outside and saw that the floor was wet. You then asked whether it had rained.) Did it rain?’
3. (C: A Maria está do outro lado da rua e não sabe se vai conseguir chamá-la:) Maria!
  ‘(C: Maria is across the street and you do not know whether you will be able to call her:) Maria!’
4. (C: O seu filho, que sempre viveu na costa, regressou de uma longa estadia no interior da Alemanha. Apesar de ele estar muito ocupado, o senhor/a senhora tem um pedido que pensa ser do seu agrado:) Anda ver o mar.
  ‘(C: Your son, who has always lived on the coast, has returned from a long stay in the interior of Germany. Although he is very busy, you have a request that you think he will appreciate:) Come to see the sea.’
5. A descida das taxas de juro foi muito elogiada.
  ‘The lowering of interest rates was highly praised.’
6. (C: Não faço ideia do que aconteceu.) Ela foi ver a Marina?
  ‘(C: I have no idea about what happened.) Did she go to see Marina?’
7. O novo presidente será eleito em Outubro.
  ‘The new president is going to be elected in October.’
8. O Álvaro, antes de partir, falou com os amigos.
  ‘Álvaro, before leaving, spoke to his friends.’
9. (C: Está a falar com a Júlia sobre os vossos amigos que vão viver para outra cidade. Tem a certeza de que vão para Lisboa, mas a sua amiga pensa que vão para Évora. Diga-lhe que não, que vão para Lisboa.) Eles não vão morar para Évora. Vão para LISBOA!
  ‘(C: You are talking with Júlia about your friends, who will move to another city. You know for sure that they are moving to Lisbon, but your Júlia thinks that they are moving to Évora. Tell her that they are moving to Lisbon.) They are not moving to Évora. They are moving to LISBON!’
10. (C: Diga de que frutas gosta mais.) Gosto de maçã, banana, uvas, laranja e tangerina.
  ‘(C: Indicate your favorite fruits.) I love apple, banana, grapes, orange, and tangerine.’
11. (C: Gostaria de saber o que se passou.) Os rapazes compraram lâminas?
  ‘(C: I would like to know what happened.) Did the boys buy blades?’
12. Espera sentada. A Maria vai demorar.
  ‘Wait seated. Maria will take long.’
13. (C: Um amigo do seu filho esqueceu-se do telemóvel em sua casa. Ele acabou de sair e o senhor/a senhora tenta ainda ir a tempo:) Valdemar!
  ‘(C: Your son’s friend has forgotten his mobile at your place. He has just left and you try to make sure you still reach him:) Valdemar!’
14. Os artistas foram sempre atraídos pelas cidades.
  ‘The artists were always attracted to the cities.’
15. (C: A sua amiga diz que a Maria e o Manuel se separaram, mas você tem a certeza de que eles se casaram. E diz à sua amiga:) Afinal, a Maria e o Manuel não se separaram. Eles CASARAM!
  ‘(C: Your friend is saying that Maria and Manuel got divorced, but you know for sure that they got married. Tell it to your friend:) After all, Maria and Manuel did not get divorced. They GOT MARRIED!’
16. Às alunas, os amigos ofereceram rosas.
  ‘To the students, their friends have offered roses.’
17. Um quadro de grande valor foi leiloado ontem.
  ‘A valuable painting was auctioned yesterday.’
18. Vi, após o tiro, uma pessoa a fugir.
  ‘I saw, after the shooting, someone running away.’
19. As alunas estrangeiras nos Açores, até onde sabemos, aceitaram vir.
  ‘The foreigner students in Azores, as far as we know, accepted to come.’
20. A inflação subiu cerca de cinco pontos no ano passado.
  ‘The inflation increased five points last year.’

### Appendix A.2. Set 2

1. A descida das taxas de juro foi muito elogiada.
  ‘The lowering of interest rates was highly praised.’
2. Às alunas, os amigos ofereceram rosas.
  ‘To the students, their friends have offered roses.’
3. (C: Está a falar com a Maria sobre os vossos amigos que foram viver para outro sítio. Tem a certeza de que mudaram para Lisboa, mas a Maria pensa que mudaram para o Sul. Diga-lhe que não, que mudaram para Lisboa.) Eles não mudaram para o Sul. Mudaram para LISBOA!
  ‘(C: You are talking with Maria about your friends, who have moved to another place. You know for sure that they have moved to Lisbon, but your Maria thinks that they have moved to the South. Tell her that they have moved to Lisbon.) They did not move to the South. They have moved to LISBON!’
4. (C: O senhor/A senhora saiu de casa e viu a rua molhada. Então, perguntou se choveu:) Choveu?
  ‘(C: You went outside and you saw that the floor was wet. You then ask whether it rained.) Did it rain?’
5. O Álvaro, após o conflito, abandonou a sala.
  ‘Álvaro, after the conflict, left the room.’
6. (C: Quer que a Marina venha para que o jantar possa ser servido. Chame-a:) Marina!!
  ‘(C: You want Marina to come so that the dinner can be served. Call her:) Marina!!’
7. A inflação subiu cerca de cinco pontos no ano passado.
  ‘The inflation increased five points last year.’
8. (C: O seu filho agarrou o jornal que o senhor/a senhora ainda não leu. Tem receio de que ele o rasgue e tenta convencê-lo a devolver o jornal:) Dá-me o jornal.
  ‘(C: Your child grasped the newspaper which you haven’t read yet. You are afraid he destroys it, so you try to convince the child to give it back to you:) Give me the newspaper.’
9. O novo presidente será eleito em Outubro.
  ‘The new president is going to be elected in October.’
10. (C: Não faço ideia do que aconteceu.) Ela foi ver a Maria?
  ‘(C: I have no idea about what happened.) Did she go to see Maria?’
11. As alunas estrangeiras em Chaves, até onde sabemos, adoram comer.
  ‘The foreigner students in Chaves, as far as we know, love to eat.’
12. Um quadro de grande valor foi leiloado ontem.
  ‘A valuable painting was auctioned yesterday.’
13. (C: O João não o/a viu e o senhor/a senhora precisava mesmo de falar com ele. Está a tentar chamá-lo:) João!
  ‘(C: João did not see you and you really need to talk to him. You are trying to reach his attention:) João!
14. Vi, atrás do bandido, um grupo de crianças.
  ‘I saw, chasing a robber, a group of children.’
15. (C: A sua amiga diz que os donos do restaurante da sua rua se separaram, mas você tem a certeza de que eles se casaram. E diz à sua amiga:) Os donos do restaurante não se separaram. Eles CASARAM!
  ‘(C: Your friend is saying that the owners of the restaurant in her street got divorced, but you know for sure that they got married. Tell that to your friend:) The owners of the restaurant did not get divorced. They GOT MARRIED!’
16. A loura cantava uma melodia maravilhosa de Espanha.
  ‘The blond girl was singing a wonderful melody from Spain.’
17. (C: Gostaria de saber o que se passou.) Os rapazes compraram lâminas?
  ‘(C: I would like to know what happened.) Did the boys buy blades?’
18. O músico compôs uma cantiga. Ela inspirou-o.
  ‘The musician has written a song. She inspired him.’
19. Os artistas foram sempre atraídos pelas cidades.
  ‘The artists were always attracted to the cities.’
20. (C: Diga que frutas comprou no mercado:) Comprei laranja, banana, limão, tangerina e cenoura.
  ‘(C: Indicate the fruits you have bought in the market:) I have bought orange, banana, lemon, tangerine, and carrot.’
